# Turicibacter faecis sp. nov., isolated from faeces of heart failure mouse model

**DOI:** 10.1099/ijsem.0.006379

**Published:** 2024-05-09

**Authors:** Yuko Imamura, Daisuke Motooka, Yuri Nakajima, Shin Ito, Masafumi Kitakaze, Tetsuya Iida, Shota Nakamura

**Affiliations:** 1 Department of Infection Metagenomics, Research Institute for Microbial Diseases, Osaka University, Suita, Japan; 2 NGS Core Facility, Research Institute for Microbial Diseases, Osaka University, Suita, Japan; 3 BIKEN-RIMD NGS Laboratory, Research Institute for Microbial Diseases, Osaka University, Suita, Japan; 4 Integrated Frontier Research for Medical Science Division, Institute for Open and Transdisciplinary Research Initiatives, Osaka University, Suita, Japan; 5 Department of Clinical Research and Development, National Cerebral and Cardiovascular Center, Suita, Japan; 6 Department of Heart Failure and Transplant, National Cerebral and Cardiovascular Center, Suita, Japan; 7 Hanwa Memorial Hospital, Osaka, Japan; 8 The Osaka Medical Research Foundation for Intractable Diseases, Osaka, Japan

**Keywords:** gut microbiota, new novel, *Turicibacter*

## Abstract

Strain TC023^T^, a Gram-positive, long, rod-shaped, spore-forming anaerobe, was isolated from the faeces of a heart failure mouse model. The strain formed greyish-white coloured colonies with a convex elevation on brain–heart infusion medium supplemented with 0.1 % sodium taurocholate, incubated at 37 °C for 2 days. Taxonomic analysis based on the 16S rRNA gene sequence showed that TC023^T^ belonged to the genus *Turicibacter*, and was closely related to *Turicibacter bilis* MMM721^T^ (97.6 %) and *Turicibacter sanguinis* MOL361^T^ (97.4 %). The whole genome of the strain has a G+C content of 37.3 mol%. The average nucleotide identity and genome-to-genome distance between TC023^T^ and *Turicibacter bilis* MMM721^T^ were 77.6 % and 24.3 %, respectively, and those with *Turicibacter sanguinis* MOL361^T^ were 75.4 % and 24.3 %, respectively. These genotypic, phenotypic, and biochemical analyses indicated that the isolate represents a novel species in the genus *Turicibacter*, and the name *Turicibacter faecis* sp. nov. is proposed. The type strain is TC023^T^ (RIMD 2002001^T^=TSD 372^T^).

## Introduction

The human gut microbiome plays an important role in cardiovascular health and disease [1,2]. The human intestine comprises 100–1000 bacterial species; however, many have not yet been isolated because they are unculturable [3,4]. Therefore, bacteria associated with cardiovascular diseases remain unclear. We previously compared the faecal microbiome between control mice and heart failure mice model developed using transverse aortic constriction (TAC) surgery [5]. Results of 16S rRNA metagenome sequencing showed that the genus *Turicibacter* was significantly increased in the faeces of TAC-treated mice compared to that in control mice. To isolate *Turicibacter* species, we screened for spore-forming bacteria from faecal samples of heart failure mice model [6,7], and subsequently isolated strain TC023^T^. Although the reference strain *Turicibacter sanguinis* MOL361^T^ is a non-spore-forming bacterium [8], *Turicibacter bilis* and some *Turicibacter* species are spore formers [6,9]. Recently, *Turicibacter bilis* MMM721^T^, *Turicibacter* sp. TA25 and 1E2 strains have been characterized as modulators of bile acids and host lipids [10,11]. In this study, we describe the phenotypic characteristics and phylogeny of the novel bacterial strain TC023^T^ belonging to the genus *Turicibacter*, and the name *Turicibacter faecis* sp. nov. is proposed.

## Isolation and culture conditions

TC023^T^ was isolated from faeces of TAC-treated mice. Briefly, a part of the stool samples stored at −80 °C was diluted in PBS, homogenized, and then incubated in freshly prepared 70 % ethanol (1 : 1 ratio) at 25 °C for 4 h under aerobic conditions [6,7]. Subsequently, the sample was washed with PBS, diluted, and spread anaerobically on brain–heart infusion (BHI) medium supplemented with 0.1 % sodium taurocholate to stimulate spore germination [6]. After incubation at 37 °C for 2 days, single colonies were picked and maintained in chocolate–blood agar media (BD).

## 16s rRNA gene phylogeny

Genomic DNA was extracted from TC023^T^ cells using a DNeasy PowerSoil kit (Qiagen). The bacterial 16S rRNA gene was amplified using the universal primers 27Fmod (5′-AGRGTTTGATCMTGGCTCAG-3′) and 1492R (5′-GGTTACCTTGTTACGACTT-3′). PCR products were sequenced using a BigDye Terminator kit and an ABI PRISM 3500xL Genetic Analyzer (Applied Biosystems, Life Technologies) [12]. Results from a blast search revealed that TC023^T^ belongs to the genus *Turicibacter*.

**Fig. 1.**
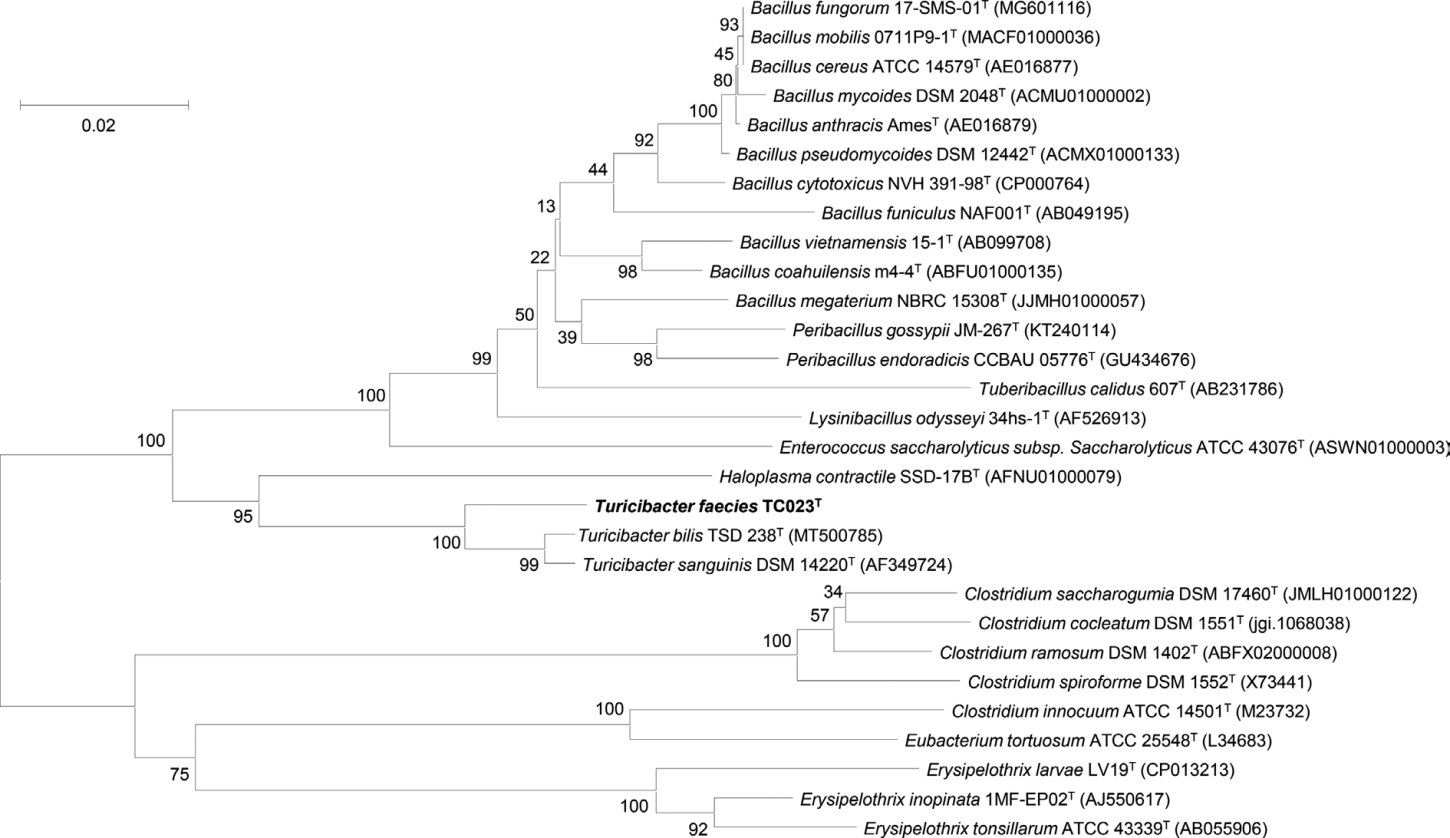
Neighbour-joining tree based on the 16S rRNA gene sequences of TC023^T^ and representative type strains belonging to the family *Erysipelotrichaceae* and *Bacillaceae*. The numbers (values>50 %) show the percentages of bootstrap derived from 1000 replications. Bar, 0.02 nt substitutions per position.

The 16S rRNA gene sequence of TC023^T^ was then compared with that of representative type strains belonging to the families *Erysipelotrichaceae* and *Bacillaceae*. Relative phylogenetic neighbours were identified using the EzTaxon database (www.ezbiocloud.net) [13]. A phylogenetic tree was reconstructed using the neighbour-joining (Fig. 1) [14], maximum-likelihood (Fig. S1, available in the online version of this article) [15], and maximum-parsimony (Fig. S2)[16] methods in mega-11 [17]. The evolutionary distances were computed using the Kimura two-parameter method [18], and the stability of clustering was determined using a bootstrap test with 1000 replicates [19]. A total of 1300 positions were included in the final dataset. The neighbour-joining phylogenetic tree showed that TC023^T^ clustered with *T. sanguinis* MOL361^T^ (DSM 14220^T^, AF349724.1). These data indicate that TC023^T^ belongs to the genus *Turicibacter*. For this comparative study, *T. sanguinis* MOL361^T^ and *T. bilis* MMM721^T^ were selected as the reference type strains. *T. sanguinis* MOL361^T^ was purchased from DSMZ (Deutsche Sammlung von Mikroorganismen und Zellkulturen).

## Genome features

The whole genome sequence of TC023^T^ was sequenced. Briefly, a genomic library was prepared using a Nextera DNA Flex Library Prep Kit (Illumina) and sequenced with 250 bp pair-end reads using the HiSeq2500 system (Illumina) [12]. Furthermore, a genomic library for Nanopore sequencing was prepared using a Ligation Sequencing Kit (ONT) and then sequenced using the MinION device, following the manufacturer’s instructions. Draft genome sequences were assembled using the Assembly Method Flye version 2.7. The whole genome of TC023^T^ was 2 431 147 bp long, with G+C content of 37.3 mol%. The assembled genome was annotated with the DDBJ Fast Annotation and Submission Tool version 1.2.6 [20]. As the results, the genome of TC023^T^ contained 2399 coding sequences (CDS) including 51 rRNA and 161 tRNA. Three gene families, *vanY*, *vanW*, and *vanT*, were predicted to be involved in resistance to glycopeptide antibiotics using the Comprehensive Antibiotic Resistance Database (https://card.mcmaster.ca/analyze/rgi). The functional annotations of the genome of strain TC023^T^ were assigned to a COG category using eggNOG-mapper version 2.1.12 (http://eggnog-mapper.embl.de/) [21] and kegg Orthology Search, KofamKOALA (www.genome.jp/tools/kofamkoala/) [22].

**Fig. 2.**
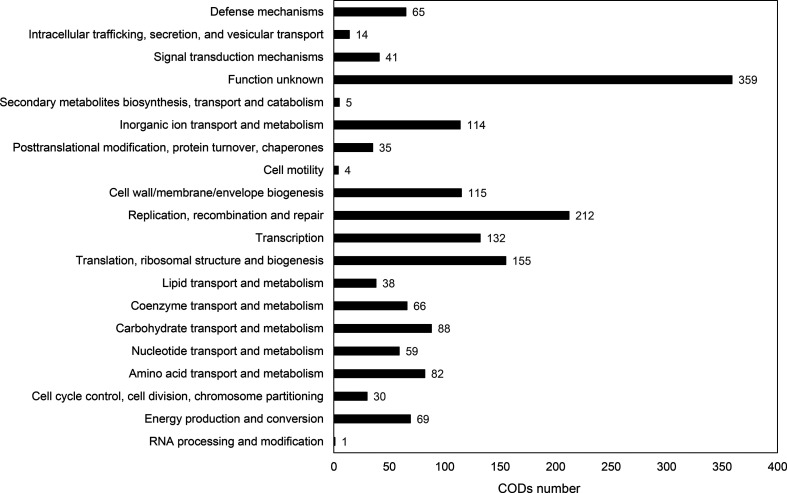
COG functional classification of the genome of TC023^T^.

As shown in Fig. 2, 1684 CDS of strain TC023^T^ allocated to a COG category were concerned with classes such as replication, recombination and repair (L; 12.6 %), translation, ribosomal structure and biogenesis (J; 9.20 %), and transcription (K; 7.84 %). Furthermore, 1166 CDS of strain TC023^T^ assigned with kegg Orthologs by KofamKOALA were involved in pathways such as carbohydrate metabolism (118 CDS), translation (77 CDS), replication and repair (66 CDS), nucleotide metabolism (59 CDS), membrane transport (55 CDS), amino acid metabolism (53 CDS), and energy metabolism (52 CDS), as shown in Fig. S3. Interestingly, the genome of TC023^T^ contained a putative *cbh* gene encoding the choloylglycine hydrolase (EC:3.5.1.24), similar to *T. sanguinis* MOL361^T^. The presence of the *cbh* gene indicates that strain TC023^T^ also metabolizes bile acids.

**Fig. 3.**
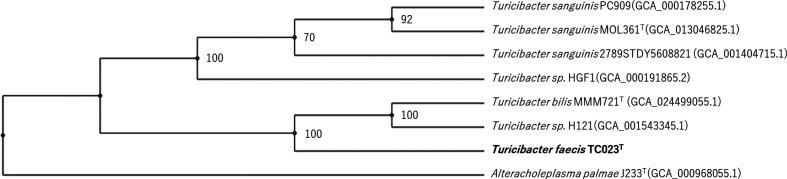
Maximum-likelihood phylogenetic tree based on genome sequences of TC023^T^ and strains belonging to the genus *Turicibacter*. Phylogeny is based on 64 concatenated gene sequences assembled using the autoMLST pipeline. *Alteracholeplasma palmae* J233^T^ (GCA_000968055.1) was used as the outgroup. The numbers (values >70 %) show the percentages of bootstrap derived from 1000 replications with iq-tree ultrafast boostrap analysis.

Genome phylogenetic analysis was conducted using the automated multi-locus species tree (autoMLST) online server (https://automlst.ziemertlab.com/) [23]. The phylogenetic tree based on 64 core genes was reconstructed using maximum-likelihood methods. As shown in Fig. 3, TC023^T^ was located in a different cluster from the species belonging to the genus *Turicibacter*.

To assess their identities, the whole genome sequence of TC023^T^ was compared with that of six other strains: *T. sanguinis* MOL361^T^ (CP053187.1: 2 999 687 bp, with 34.4 mol% G+C content), *T. bilis* MMM721^T^ (CP071249.1: 2 786 784 bp, with 34.6 mol% G+C content), *T. bilis* PIG517 (CP071251.1: 2 663 111 bp, with 34.6 mol% G+C content), *T. bilis* ISU324 (CP071250.1: 2 751 004 bp, with 34.5 mol% G+C content), *Turicibacter* sp. H121 (CP013476.1: 2 622 031 bp, 34.8 mol% G+C content), and *Turicibacter* sp. TJ11 (CP069349.1: 2 651 593 bp, with 34.0 mol% G+C content) [24,25]. Average nucleotide identity (ANI) values were analysed using the JSpeciesWS Online Service (http://jspecies.ribohost.com/jspeciesws/) [26], orthologous ANI (OrthoANI) values were calculated using the EzBioCloud web service (www.ezbiocloud.net/tools/ani) [27], and digital DNA–DNA hybridization (dDDH) with *in silico* methods was estimated using the Genome-to-Genome Distance Calculator 2.1 web service (http://ggdc.dsmz.de/me.php) [28]. As shown in Table 1, the ANIb and ANIm values of TC023^T^ with reference to the other *Turicibacter* strains were 74.6–77.1 % and 85.6–86.2 %, respectively. Moreover, the orthoANI and dDDH values were 75.4–77.8 % and 24.3–25.0 %, respectively. These data confirm that strain TC023^T^ represents a novel species in the genus *Turicibacter*.

**Table 1.** Genome-based comparison between TC023^T^ and its closely related strains from the genus *Turicibacter* Strain 1, *Turicibacter sanguinis* MOL361^T^ (CP053187.1); 2, *Turicibacter bilis* MMM721^T^ (CP071249.1); 3, *Turicibacter bilis* PIG517 (CP071251.1); 4, *Turicibacter bilis* ISU324 (CP071250.1); 5, *Turicibacter* sp. H121 (CP013476.1); 6, *Turicibacter* sp. TJ11(CP069349.1).

Characteristic	1	2	3	4	5	6
ANIb (%)	74.6	76.8	77	76.9	77.1	76.4
ANIm (%)	85.6	85.7	85.7	85.9	85.7	86.2
Tetra	0.95	0.96	0.96	0.96	0.96	0.95
OrthoANI (%)	75.4	77.6	77.4	77.6	77.8	77.0
G+C content difference (mol%)	2.85	2.63	2.63	2.71	2.39	2.96
dDDH (%)	24.3 [22.0–26.8]	24.3 [22.0–26.8]	24.0 [21.7–26.5]	24.4 [22.1–26.9]	24.4 [22.1–26.8]	25.0 [22.7–27.5]

## Morphology, physiology and chemotaxonomy

TC023^T^ cell morphology was observed under light and transmission electron microscopes (Fig. S4). Cells were propagated on chocolate–blood agar medium at 37 °C for 24 h. Gram staining was performed using a it (Muto Pure Chemicals Co.). Bacterial growth at different temperatures (25, 28, 32, 37, 42, or 45 °C) was examined using chocolate–blood agar medium for 7 days. The effect of pH was determined on BHI agar plates using the pH range 6.0–9.0 with 0.5 pH unit intervals (adjusted with 1 M HCl or NaOH). Growth on different media (BHI, BHI with 0.1 % sodium taurocholate, chocolate blood, sheep blood, MacConkey, or Reasoner's 2A) was also monitored for 7 days at 37 °C.

Oxidase activity was evaluated using a cytochrome oxidase test strip (Nissui), and catalase activity was assessed by observing bubble production after dropping a 3 % hydrogen peroxide solution. Both oxidase activity and catalase activity of strain TC023^T^ were negative, similar to *T. sanguinis* DSM 14220^T^ and *T. bilis* MMM721^T^. Other physiological properties and enzyme activities were explored using the API ZYM, API 50CH, API 20A, and API rapid ID 32A systems, following the manufacturer’s instructions (bioMérieux). The results showed that TC023^T^ harboured α-glucosidase and α-galactosidase, and fermented aesculin, maltose, and 5-ketogluconate, similar to *T. sanguinis* DSM 14220^T^ (Tables 2 and S1). In contrast, TC023^T^ showed physiological characteristics different from thosr *T. bilis* MMM721^T^.

**Table 2.** Biochemical characteristics of TC023^T^ and its closely related type strains Strain 1, TC023^T^; 2, *Turicibacter sanguinis* DSM 14220^T^; 3, *Turicibacter bilis* MMM721^T^. +, Positive; –, negative; ±, variable reaction. The characteristics of *T. bilis* MMM721^T^ were obtained from a study by Maki *et al.* [9].

Characteristic	1	2	3
Isolation source	Mice faeces	Human blood	Chicken eggshell
Morphology	Bacilli chains	Bacilli chains	Bacilli chains, coccoid cluster
Spore forming	+	–	+
Optimum growth temperature (°C)	37	37	42
Optimum growth pH	7.5	7.5	7.5
Motility	–	–	–
Catalase activity	–	–	–
Enzyme activities:			
α-Glucosidase	+	+	–
α-Galactosidase	+	+	–
Arginine arylamidase	–	–	+
Glycine arylamidase	–	+	–
Serine arylamidase	–	+	–
Carbon utilization:			
Gelatin	–	+	+
Aesculin	+	±	±
Maltose	+	+	–
5-Keto-gluconate	+	±	–
Genome length (Kb)	2431	2999	2717
DNA G+C content (mol%)	37.3	34.4	34.4

Fatty acids were prepared and analysed by Techno Suruga Laboratory Co., Ltd. (Shizuoka, Japan) using the Sherlock Microbial Identification System with the moore6 database (version 6.0, midi), as previously described [29]. The major fatty acids (>10 %) in strain TC023^T^ were C_16 : 0_ and C_18 : 0_, and the minor fatty acids were C_14 : 0_, C_17 : 0_, and C_18 : 1_ ω9*c*, similar to *T. sanguinis* DSM 14220^T^ and *T. bilis* MMM721^T^ (Table 3). Summed feature 10 (C_18 : 1 _ω7*c* and/or unknown 17.834) was the major fatty acid in strain TC023^T^ and *T. sanguinis* DSM 14220^T^, but not in *T. bilis* MMM721^T^.

**Table 3.** Cellular fatty acid composition of TC023^T^ and its closely related type strains Strain 1, TC023^T^, 2, *Turicibacter sanguinis* DSM 14220^T^, 3, *Turicibacter bilis* MMM721^T^. Fatty acids (>1 %) are shown, and total fatty acid content >10 % is highlighted with bold text. The bar indicates not detected. The component values for *T. bilis* MMM721^T^ were obtained from Maki *et al.* [9].

Fatty acids (%)	1	2	3
C_14 : 0_	2.94	1.70	2.84
C_16 : 1_ ω7*c*	3.35	0.85	1.41
C_16 : 0_	**48.1**	**39.6**	**54.1**
C_17 : 0_	1.66	5.45	1.80
C_18:1_ ω9*c*	6.22	2.80	5.78
C_18:1_ ω5*c*	1.12	0.97	1.00
C_18 : 0_	**10.6**	**14.7**	**11.8**
Summed feature:			
C_18:1_ ω7*c* and/or unknown 17.834	**19.1**	**27.6**	–

## Description of *Turicibacter faecis* sp. nov.

*Turicibacter faecis* (fae’cis. L. gen. n. *faecis*, of faeces, the isolated source of the strain).

Cells are Gram-positive, catalase-negative, oxidase-negative, anaerobic, spore-forming, non-motile, and irregularly rod-shaped (0.5–1.5×0.7–5.0 µm). Colonies on chocolate–blood agar media appear greyish-white with convex elevations. The type strain grows at 32–42 °C (optimal at 37 °C) and pH 6.5–8.0 (optimal at pH 7.5). Cells grow well on BHI medium with 0.1 % sodium taurocholate, chocolate, and sheep blood agar media, but not in BHI, MacConkey, or Reasoner's 2A media. C_16 : 0_, C_18 : 0_, and summed feature 10 (C_18 : 1_ ω7*c*) are the major fatty acids. In the API ZYM assays, the strain is positive for esterase (C4), acid phosphatase, naphthol-AS-BI phosphohydrolase, α-galactosidase, β-galactosidase, alkaline phosphatase, esterase lipase (C8), and β-glucosidase; and negative for lipase (C14), leucine arylamidase, valine arylamidase, cystine arylamidase, trypsin, α-chymotrypsin, β-glucuronidase, α-glucosidase, *N*-acetyl-β-glucosaminidase, α-mannosidase, and α-fucosidase. Further, it is positive for α-galactosidase, β-galactosidase, α-glucosidase, glutamic acid decarboxylase, alkaline phosphatase, and pyroglutamic acid allyl amidase in the API rapid 32A strips, but is negative for β-glucuronidase. Glucose, lactose, sucrose, maltose, and raffinose are acidified, whereas d-mannitol, d-xylose, l-arabinose, glycerol, d-mannose, melezitose, d-sorbitol, l-rhamnose, and trehalose are not acidified in the API20A tests. The type strain is also positive for aesculin hydrolysis, but negative for gelatin hydrolysis, arginine hydrolysis, nitrate reduction, indole production, and urea activity. In the API 50CH tests, cells assimilate only d-ribose, aesculin ferric citrate, d-tagatose, and 5-ketogluconate.

The type strain, TC023^T^ (=RIMD 2002001^T^=TSD 372^T^), was isolated from a faecal sample of heart failure model mice. The genome has a G+C content of 37.3 mol%. The 16S rRNA gene and whole genome sequence of TC023^T^ are deposited with GenBank accession numbers LC719463 and AP028127, respectively.

## supplementary material

10.1099/ijsem.0.006379 Uncited Supplementary Material 1.

## Abbreviations

ANI: average nucleotide identity
BHI: brain–heart infusion
CDS: coding sequences
dDDH: digital DNA–DNA hybridization
DMA: dimethyl acetals
TAC: transverse aortic constriction
