# Supplementary material for: Turicibacter faecis sp. nov., isolated from faeces of heart failure mouse model
Source: Int J Syst Evol Microbiol. 2024 May 9;74(5):006379. doi: 10.1099/ijsem.0.006379 (PMC11165905; doi:10.1099/ijsem.0.006379)
Supplement: Uncited Supplementary Material 1. [file ijsem-74-06379-s001.pdf]

Supplemental Materials

Turicibacter faecis sp. nov., isolated from feces of heart failure mouse model

Yuko Imamura[1, *], Daisuke Motooka[1, 2, 3, 4], Yuri Nakajima[5], Shin Ito[5, 6], Masafumi Kitakaze[7, 8], Tetsuya Iida[1, 3] and Shota Nakamura[1, 2, 4]

[1] Department of Infection Metagenomics, Research Institute for Microbial Diseases, Osaka University, Suita, Japan

[2] NGS Core Facility, Research Institute for Microbial Diseases, Osaka University, Suita, Japan

[3] BIKEN-RIMD NGS Laboratory, Research Institute for Microbial Diseases, Osaka University, Suita, Japan

[4] Integrated Frontier Research for Medical Science Division, Institute for Open and Transdisciplinary Research Initiatives, Osaka University, Suita, Japan

[5] Department of Clinical Research and Development, National Cerebral and Cardiovascular Center, Suita, Japan

[6] Department of Heart Failure and Transplant, National Cerebral and Cardiovascular Center, Suita, Japan

[7] Hanwa Memorial Hospital, Osaka, Japan; [8] The Osaka Medical Research Foundation for Intractable Diseases, Osaka, Japan

Corresponding author: Yuko Imamura, E-mail: ydoiguchi@gen-info.osaka-u.ac.jp

Supplementary Fig.S1. Maximum-likelihood tree based on 16S rRNA gene sequences, showing the relationships of strain TC023^T with representative type strains belonging to the family *Erysipelotrichaceae* and *Bacillaceae*. The numerals (values >50% are noted) show the percentages of bootstrap derived from 1000 replications. The scale bar equals 0.05 changes per nucleotide position.

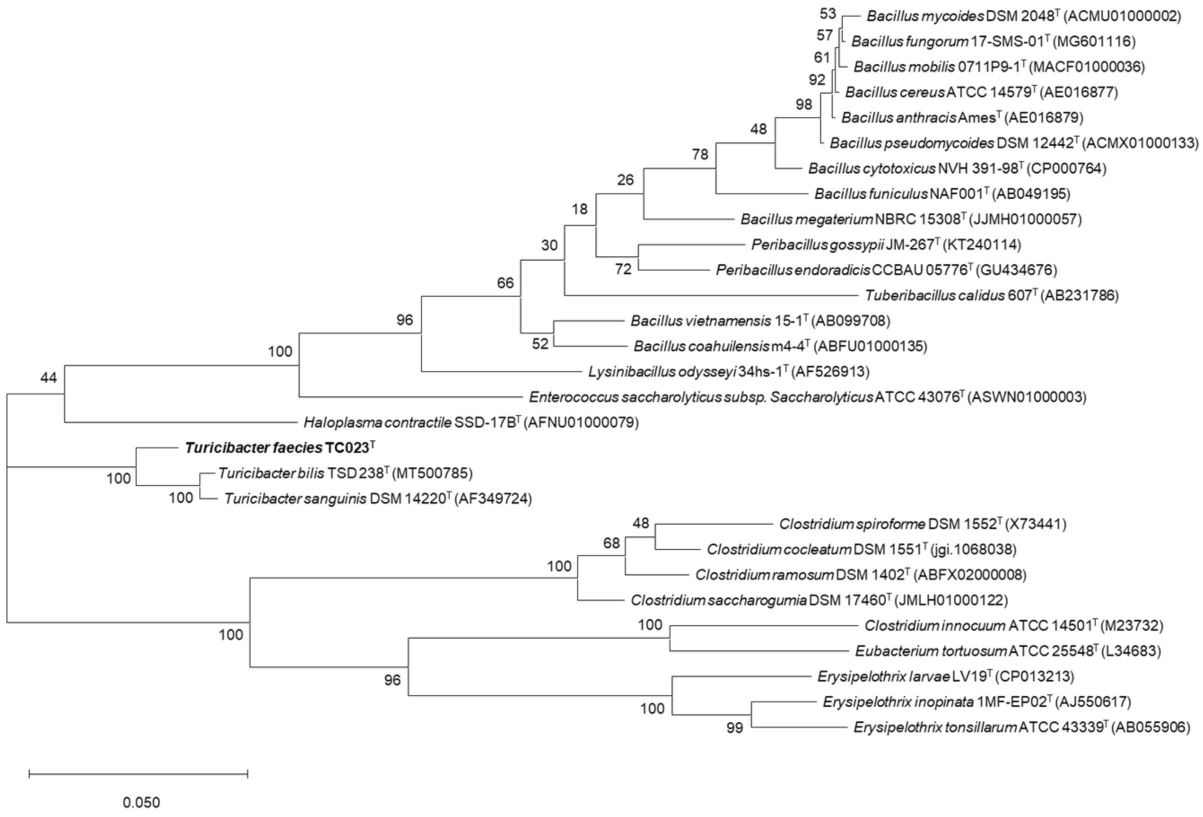

Supplementary Fig.S2. Maximum-parsimony tree based on 16S rRNA gene sequences, showing the relationships of strain TC023^{T} with representative type strains belonging to the family *Erysipelotrichaceae* and *Bacillaceae*. The numerals (values >50% are noted) show the percentages of bootstrap derived from 1000 replications. The scale bars indicate the numbers of nucleotide changes over the whole sequences.

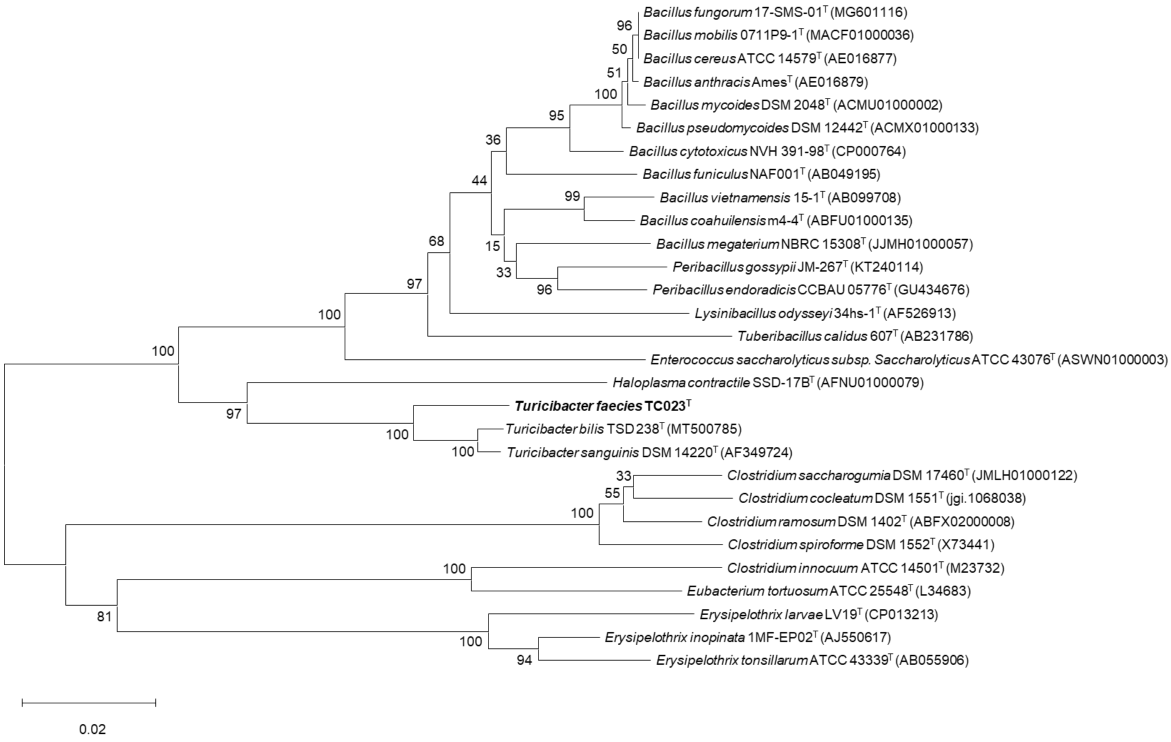

Supplementary Fig.S3. KEGG functional classification in the genome of $TC023^T$.

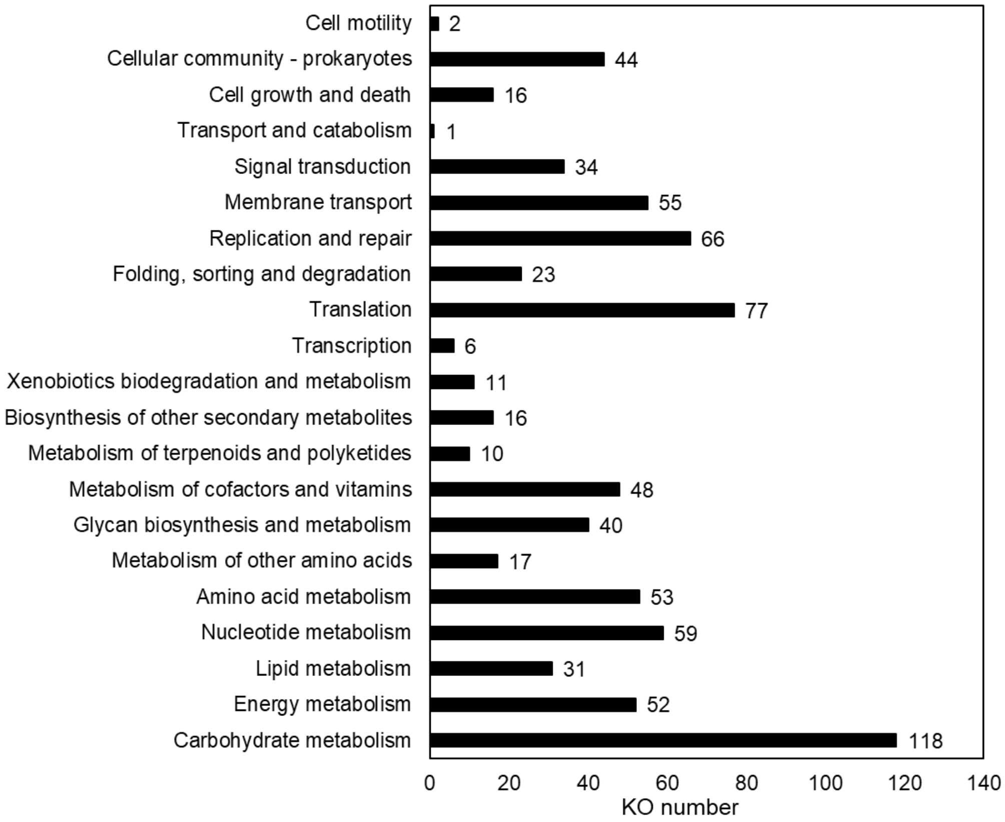

Supplementary Fig.S4. Transmission electron microscope image of a negatively stained cell of strain TC023^{T}. Bar, 1 μm.

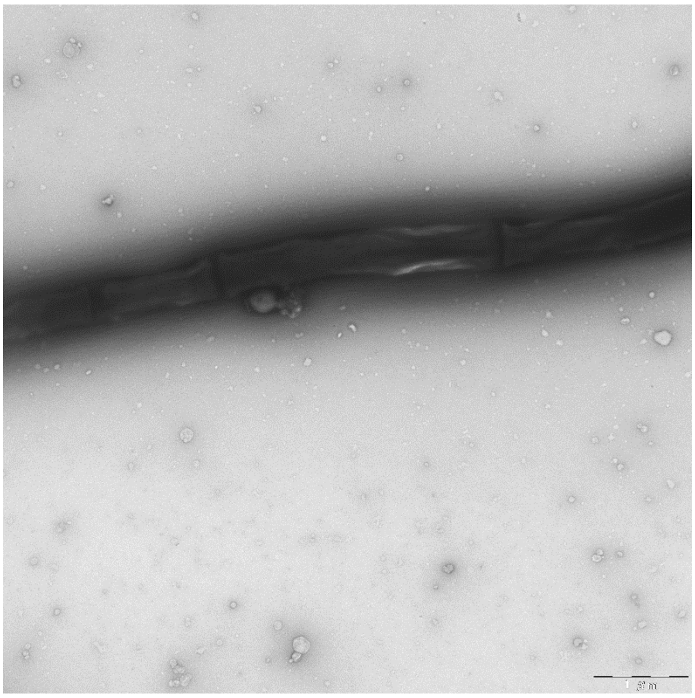

Supplementary Table S1. Phenotypic characteristic of TC023^T and *Turicibacter Sanguinis* DSM 14220^T. Strains: 1, TC023^T, 2, *Turicibacter Sanguinis* DSM 14220^T. All data are from this study. +, positive; -, negative; w, weak reaction.

**Characteristic**	**1**	**2**
Isolation source	Mice feces	Human blood
Cell shape	Long rods	Long rods
Spore presence	+	-
Catalase activity	-	-
Oxidase activity	-	-
**Enzyme activities**		
Alkaline phosphatse	+	+
Esterase	+	w
α-Galactosidase	+	+
β-Galactosidase	+	+
α-Glucosidase	+	+
β-Glucosidase	-	-
N-acetyl-β-glucosaminidase	-	-
Glutamic acid decarboxylase	+	+
Pyroglutamic acid allyl amidase	+	+
**Carbon utilization**		
D-Ribose	+	w
Esculin ferric citrate	+	+
D-Tagatose	w	+
5-Keto-Gluconate	+	+
**Acid production from**		
Glucose	+	+
D-Mannitol	-	w
Lactose	+	+
Sucrose	+	+
Maltose	+	+
Salicin	w	+
D-Xylose	-	-
L-Arabinose	-	-
D-Cellobiose	w	+
D-Mannose	-	+
D-Melezitose	-	+
D-Raffinose	+	+
D-Sorbitol	-	+
L-Rhamnose	-	w
D-Trehalose	-	+
